# The birth of embryonic pluripotency

**DOI:** 10.1098/rstb.2013.0541

**Published:** 2014-12-05

**Authors:** Thorsten Boroviak, Jennifer Nichols

**Affiliations:** 1 Department of Biochemistry, University of Cambridge, Cambridge CB2 1GA, UK; 2 Department of Physiology, Development and Neuroscience, University of Cambridge, Cambridge CB2 3DY, UK; 3 Wellcome Trust–Medical Research Council Cambridge Stem Cell Institute, University of Cambridge, Tennis Court Road, Cambridge CB2 1QR, UK

**Keywords:** cleavage, totipotency, trophoblast, epiblast, primitive endoderm, pluripotency

## Abstract

Formation of a eutherian mammal requires concurrent establishment of embryonic and extraembryonic lineages. The functions of the trophectoderm and primitive endoderm are to enable implantation in the maternal uterus, axis specification and delivery of nutrients. The pluripotent epiblast represents the founding cell population of the embryo proper, which is protected from ectopic and premature differentiation until it is required to respond to inductive cues to form the fetus. While positional information plays a major role in specifying the trophoblast lineage, segregation of primitive endoderm from epiblast depends upon gradual acquisition of transcriptional identity, directed but not initiated by fibroblast growth factor (FGF) signalling. Following early cleavage divisions and formation of the blastocyst, cells of the inner cell mass lose totipotency. Developing epiblast cells transiently attain the state of naive pluripotency and competence to self-renew *in vitro* as embryonic stem cells and *in vivo* by means of diapause. This property is lost after implantation as the epiblast epithelializes and becomes primed in preparation for gastrulation and subsequent organogenesis.

## 1. Background

Mammalian preimplantation development combines establishment of a small population of founder cells for the fetus with early differentiation of extraembryonic tissues required to facilitate implantation, patterning and nutrition. The transcriptional and translational machinery becomes activated to institute self-sufficient cell populations from the maternally dominated zygote. Once established, the embryonic lineage must be protected from premature differentiation to remain susceptible to subsequent positional and temporal patterning in order to orchestrate formation of all the tissues in the body. This property is known as naive pluripotency [1]. An interesting and biomedically relevant asset of the murine preimplantation epiblast is its ability to remain undifferentiated and proliferate when explanted into appropriate culture conditions in the form of embryonic stem (ES) cells. In this chapter, we review the current knowledge of how this intriguing state of ‘naive’ pluripotency is acquired *in vivo*.

## 2. Totipotency is a unique property of cleavage stages

**Figure 1.**
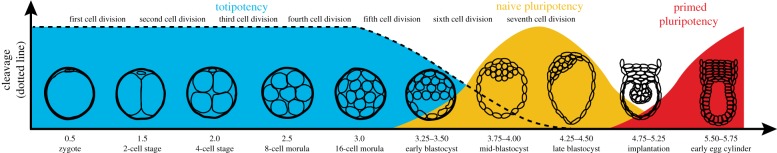
Overview of embryonic potential in relation to developmental stage from zygote to egg cylinder. Cleavage is indicated by the dotted line and correlates with totipotency (blue). Naive pluripotency (yellow) is established at the mid-blastocyst stage and persists until implantation. The terms totipotency, naive pluripotency and primed pluripotency (red) apply to the embryonic lineage only. (Online version in colour.)

The fertilized egg is capable of producing all embryonic as well as extraembryonic lineages. This distinctive ability is referred to as totipotency. However, preparation for totipotency in mammals begins long before fertilization. In mouse, the volume of the developing oocyte increases approximately 500-fold during intra-ovarian growth. Continuous transcription of the maternal genome yields around 100 pg messenger RNA in mature oocytes, with some transcripts remaining dormant in order to become activated after fertilization. By contrast, sperm has lost most of its organelles during spermatogenesis in exchange for motility and therefore depends on the egg to boot the embryonic genome. After fertilization, maternal proteins and transcripts pave the way to the first major wave of transcription at the 2-cell stage in mouse [2] and continue to play a role in the initial stages of development. The first five cell cycles, commonly referred as cleavage divisions, are characterized by a predominant S-phase, while G-phases are present but short and variable [3,4]. Cleavage occurs in the absence of cellular growth or increase in total cell mass [5] and strictly depends on the large cytosolic compartment of the fertilized egg (figure 1). Cells generated by cleavage divisions are referred to as blastomeres. At the 2-cell stage, blastomeres retain the ability to form an entire conceptus, evident from the formation of identical twins and demonstrated by the production of viable offspring in mice after destruction of one of the two blastomeres [6,7]. However, monozygotic twins are a rare phenomenon and recent work revealed that a minimum of four preimplantation epiblast cells has to be established for successful normal development [8]. Moreover, the efficiency for monozygotic twins can be increased by modulation of fibroblast growth factor (FGF) and Wnt signalling [8].

Individual blastomeres of the 4- and 8-cell stage can also progress in development and form trophoblastic vesicles as well as small blastocysts [9], which can implant in the uterus when transferred into a synchronized recipient [10]. However, the resultant decidua mostly contained trophoblast giant cells and on only one occasion a retarded embryo [10], suggesting that single 1/4 and 1/8 blastomeres are not capable of producing an entire fetus on their own. Experiments in which isolated blastomeres from 4- and 8-cell stages were aggregated with host blastomeres from another embryo have shown that they are able to differentiate into both trophectoderm and inner cell mass (ICM) and yield viable pups [11]. Thus, their failure to form a normal fetus in isolation is most probably due to inadequate numbers of cells, rather than a restriction in developmental potential. The fact that all blastomeres derived from the 4- and 8-cell stage contribute to both extraembryonic and embryonic lineages demonstrates their principal equipotency.

## 3. Compaction controls the first lineage decision

One of the most intriguing questions in developmental biology is how lineage identity can be acquired from apparently uniform 8-cell blastomeres. A possible answer could be that early blastomeres might not be as identical as they appear. Several studies have highlighted differences between individual blastomeres, including differential methylation patterns [12], potency under the influence of certain conditions [13] and transcription factor kinetics [14]. However, the majority of blastomeres retain embryonic and extraembryonic potential and differentiate based on their position within the 8- to 16-cell embryo [15].

How do blastomeres ‘sense’ their position? A crucial event preceding the first lineage decision is compaction, which occurs at the late 8-cell stage, at around embryonic day (E) 2.75. During compaction, the blastomeres increase their intercellular interactions, thereby providing the essential spatial queues for the first lineage decision in the mammalian embryo. This allows the establishment of differential compartments. Initially formulated as the ‘inside–outside’ hypothesis [9], subsequent experiments have confirmed that the spatial location of individual blastomeres is instructive for their subsequent lineage allocation [11]. In normal development, the outer cells of the morula become biased towards the first extraembryonic lineage, the trophectoderm. Trophectoderm is required for implantation and subsequently will give rise to the placenta, an extraembryonic organ pivotal for nourishment, detoxification and patterning of the developing fetus [16]. By contrast, cells located in the inside tend to form the ICM of the early blastocyst. ICM cells maintain expression of the POU-domain transcription factor Oct4 (Pou5f1), which is downregulated in outside cells. In the absence of Oct4, the inside cells fail to maintain their identity and differentiate into trophectoderm [17]. Using ES cells, it has been shown that Oct4 acts cooperatively with Sox2 to induce expression of several pluripotency genes, including FGF4 [18] and Nanog [19]. In line with this, embryo profiling at single-cell resolution revealed Sox2 and Id2 as the earliest markers of inner and outer cells, respectively, specifically upregulated at the 16- and 32-cell stage [20].

During compaction, intercellular adhesion depends on E-cadherin [21], and outside cells acquire apical–basal polarity by asymmetric localization of the polarity proteins atypical protein kinase C [22], Par3 [23] and the actin-associated protein ezrin [24]. Interference with polarity regulators by RNAi microinjection perturbs trophectoderm development [23,25], placing polarization upstream of the first lineage decision in the embryo, but downstream of the ‘inside–outside’ spatial location of the individual blastomeres. This polarity is given particular consideration in the ‘polarity’ model of the first lineage decision during cleavage [26]. Key to the model is that the embryo becomes radially polarized at the compacted morula stage, originally discovered by the formation of an external microvillous pole on each blastomere. The model then suggests that this polarity can be inherited during the next (fourth) cleavage division, as most blastomeres will give rise to one polar cell, which inherits the outside surface, and one apolar cell, completely engulfed by other blastomeres. The remaining cells divide symmetrically by splitting the microvillous apical domain, thereby producing two polarized daughters, both of which harbour an outside surface. This model is consistent with the morphology of an average 16-cell embryo, which contains approximately 10–14 outer, polar and 2–6 inner, apolar cells [26–28]. It is worth pointing out that cell fates are not determined in the initial stages of blastocyst formation, as outside 16-cell blastomeres still retain the potential to become ICM at robust frequencies when put into an earlier stage. Moreover, aggregations of purely outer cells can form a new embryo, capable of development in the uterus [15], providing further evidence for the persistence of totipotency in a substantial proportion of blastomeres at this stage.

## 4. Hippo signalling conveys cellular polarity into lineage-specific gene expression

**Figure 2.**
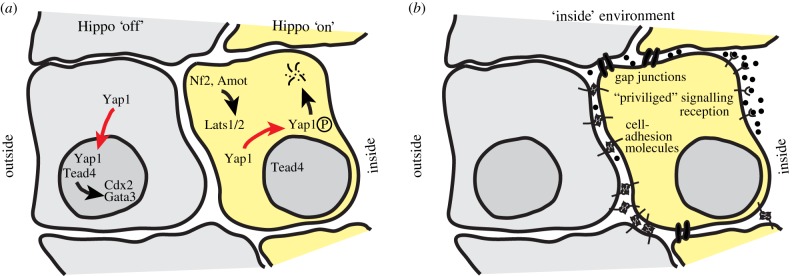
Hippo signalling and an ‘inside’ requirement for the establishment of the embryonic lineage. (*a*) Schematic of Hippo signalling activities in inside and outside cells of a 16-cell morula. (*b*) Hippo signalling alone is not sufficient for embryonic lineage formation. Potential signalling activities mediated by the inside environment are outlined. (Online version in colour.)

A key question in the context of embryonic lineage specification is how ‘inside’ or ‘outside’ spatial information is translated into transcriptional programmes. These are established by lineage-specific master regulators, including Cdx2 and Gata3 for trophectoderm versus Oct4, Sox2 and Nanog in the ICM [20,29–32]. Cdx2 null embryos are capable of trophectoderm specification but require Cdx2 for morphological integrity, subsequent development and implantation [33]. The discovery that loss of Tead4 leads to complete failure in blastocyst cavity formation places it upstream of the trophectoderm transcriptional network [34,35]. Intriguingly, Tead4 activity is not mediated by specific expression, but rather by intracellular localization regulated by the Hippo signalling cascade [36]. Hippo signalling is a highly evolutionarily conserved pathway, which, in the context of the mouse embryo, integrates positional information into lineage specification (figure 2*a*). In mammalian embryos, Hippo signalling is active in inside cells when Lats1/2 phosphorylates the Yorki homologues Yap1 and Wwtr1 [36]. Phosphorylated Yap1 is excluded from the nucleus and degraded. Consequently, Yap1 cannot act as co-activator for Tead4, resulting in failure to induce the trophectodermal programme via expression of Gata3 and Cdx2 [36,37]. In outside cells, Lats1/2 remains inactive, allowing Yap1 to enter the nucleus, and in combination with Tead4, to prime the cell towards trophectoderm. Consistent with this, reduction of Lats1/2 in the early preimplantation embryo prevents ICM lineage formation [38]. Recent work suggests that Lats1/2 activity is controlled by Nf2, which promotes interaction between the adherens junctions and Amot, another regulatory component of Hippo signalling in early mouse development [39,40].

## 5. Inner cell mass specification requires an ‘inside’ environment

**Figure 3.**
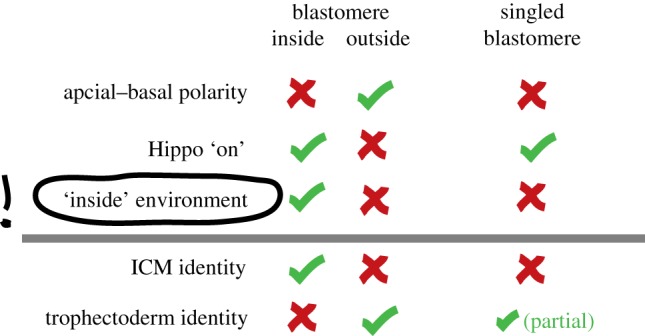
Summary of the cellular characteristics of blastomeres in the embryo and singled blastomeres grown in isolation [41]. Although singled blastomeres lack apical–basal polarity and active Hippo signalling (similar to ‘inside’ cells destined to become ICM), they partially recapitulate trophectoderm identity. This suggests an important role for the ‘inside’ environment in the establishment of the embryonic lineage, which is lacking in singled blastomeres. (Online version in colour.)

Hippo signalling alone is not sufficient to control entirely the first lineage decision. Nf2 overexpression fails to alter Yap localization, probably because of other missing components in outside cells [40]. Knockdown of Lats1/2 leads to ectopic Cdx2 expression in the ICM, but concurrent with persistent expression of Oct4 and Nanog, suggesting incomplete conversion of inner cells to bona fide trophectoderm [38]. Thus, additional information may be required to establish ICM fate [38], besides the lack of an apical domain. For instance, inside cells may use gap junction-mediated intercellular communication and adherens junctions, potentially leading to cytoskeletal alterations and signalling activities via focal adhesion kinases (figure 2*b*). Furthermore, inside cells may reside in a privileged position to receive signalling molecules. Considering the confined intercellular space, even small amounts of secreted ligand would be experienced at higher concentrations inside. Finally, inside cells may be exposed to a specific ‘basal’ environment as the result of asymmetrical protein localization in outside cells. Functional evidence for an ‘inside’ requirement in addition to Hippo signalling comes from blastomeres grown in isolation [41]. Blastomeres were separated after each of the first five cell divisions (1/32), subjected to lineage marker expression profiling, and compared to ICM and trophectoderm cells. Although their expression pattern was distinct from both, it was closer to trophectoderm than ICM [41], corroborating the requirement for an inside environment for ICM specification. This study also demonstrated that singled blastomeres preferentially contribute to trophectoderm in morula aggregations [41]. Interestingly, Hippo signalling is induced in singled blastomeres, suggesting that loss of apical–basal polarity is insufficient to adopt ICM fate (figure 3). In support of this, blastomeres have the ability to give rise to functional trophectoderm when transferred into a recipient female as single cells, but do not form embryonic tissues [10]. Embryos at the 4-cell stage denuded of the zona pellucida can rearrange their cells into various configurations during culture. Those adopting a linear configuration, where intercellular interactions are low, result in blastocysts with significantly fewer ICM cells [42] and exhibit inferior development when transferred into the uterus, compared with tetrahedral configurations, where intercellular interactions are maximized [43]. Single 1/4 and 1/8 blastomeres give rise to ‘blastocysts' at frequencies of 40% and 15%, respectively, while the number of empty trophoblastic vesicles increases [9]. Collectively, these data suggest that blastomeres grown in isolation, despite the loss of apical–basal polarity, become biased towards trophectoderm and fail to enter the embryonic lineage.

## 6. Totipotency is gradually lost in the inner cell mass

A widely understated characteristic of the early ICM is its totipotency (figure 1). In this context, we define totipotency as the ability of a cell to contribute to all embryonic and extraembryonic lineages. Clearly, this totipotency is different from the ‘absolute’ totipotency of the zygote, which is capable of forming an entire organism from one cell. However, ICMs isolated from early blastocysts have the ability to regenerate trophectoderm, resulting in miniature blastocysts [44,45], and can differentiate into trophectoderm when explanted *in vitro* [46,47]. Furthermore, they can contribute to trophectoderm in ICM–morula aggregations [48]. Aggregation of several isolated ICMs can compensate for cell numbers and regulate their combined size to produce apparently normal blastocysts. Strikingly, more than one-third of these aggregates give rise to complete egg cylinders upon transfer into recipient female mice [48]. A recent study tested the developmental potential of ICM cells at various blastocyst stages and found that early ICM cells frequently contribute to trophectoderm when injected into a morula, confirming the previously observed developmental plasticity [49]. This ability is gradually lost after E3.5 when the ICM cell number exceeds approximately 16–19 cells [48,49], concomitant with the second lineage decision in the mouse embryo: the segregation of pluripotent epiblast and primitive endoderm (PrE).

## 7. The second lineage decision: partitioning the inner cell mass into preimplantation epiblast and primitive endoderm

With the advent of accessible custom-made antibodies and fluorescent lineage reporters, the process of PrE and epiblast segregation has been interrogated and is reviewed in great detail elsewhere [50–54]. Here, we outline the differences of the second lineage decision compared to the position-dependent induction of trophectoderm discussed above.

The early PrE marker, Gata6, is initially co-expressed with the pluripotent epiblast marker, Nanog, in the early ICM [55]. Consistent with this, a recent study has shown that at the early blastocyst stage (32-cell), the transcriptome of individual ICM cells is indistinguishable [56]. However, within the next couple of hours of development, small transcriptional changes become progressively manifested and the cells subsequently segregate into two discrete populations [20,56]. In mouse, this process is mainly driven by FGF signalling [57,58]. A cardinal feature of epiblast cells is their temporal unresponsiveness to FGF signalling during the segregation process. Transcriptome analysis of early ICM and epiblast cells has shown that FGFR2, FGFR3 and FGFR4 are specific to the PrE lineage, while FGFR1 is expressed in all cells [56]. Loss of FGF4, FGFR2 or its downstream mediator, Grb2, ablates PrE formation [57,59,60], whereas loss of the other FGF receptors exhibits phenotypes at later stages of development. Therefore, FGFR2 is the essential receptor for PrE specification. However, initiation of the PrE transcriptional programme does not exclusively depend on FGF signalling; embryos completely devoid of FGF4 exhibit mosaic expression of early markers of PrE, such as Gata6 and Sox17 [61].

**Figure 4.**
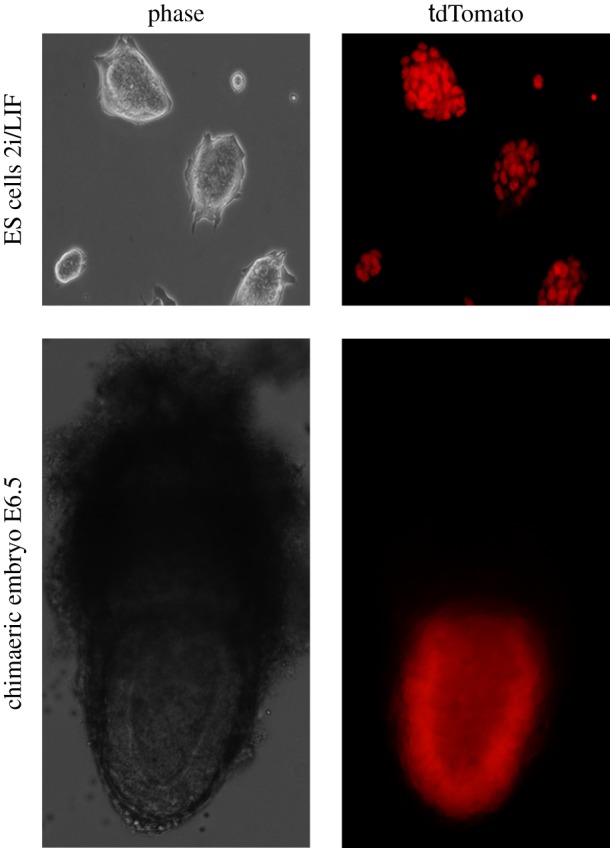
ES cells taking over the host embryo. Fluorescently labelled (tdTomato) mouse ES cells, grown under serum- and feeder-free 2i/LIF culture conditions (upper panel), were injected into non-labelled host morulae. The embryonic compartment (postimplantation epiblast) of the resulting chimaeras apparently consists entirely of donor-derived cells (lower panel). Left images: bright field; right images: fluorescence. (Online version in colour.)

In line with the genetic evidence, exogenous modulation of FGF signalling in culture from the mid-blastocyst stage or earlier influences ICM cell fate [62–64]. Inhibition of the FGF/Erk pathway with synthetic inhibitors directs ICM cells to become epiblast, whereas supplementation with exogenous FGF4 or FGF2 leads preferentially to PrE. The high concentrations of ligand required to accomplish this lineage switch seem somewhat perplexing, but these may approximate in real terms to the high expression levels of FGF4 secreted by epiblast progenitors [56,65] acting over a comparatively short range within the ICM. Evidence that physiological levels of FGF4 can direct immature ICM cells to become PrE is provided by formation of chimaeras between ES cells and cleavage stage embryos. During the aggregation process, ES cells will preferentially occupy the inside compartment of the embryo, displacing the host cells. The resulting fetus is frequently composed entirely of ES cell derivatives [66], whereas the extraembryonic endoderm almost exclusively originates from the host embryo [67] (figure 4). Once initiated, the inverse correlation of FGF4 in presumptive epiblast cells and its cognate receptor, FGFR2, in PrE precursors increases in order to reinforce the differential identity of the two lineages [20]. By the time the embryo is ready to implant in the uterus, the cells are irreversibly committed to their respective lineages [49,68].

The important question of how the symmetry of transcriptional regulators is broken in the early ICM is still debated. It has been suggested that stochastic fluctuations in gene expression, followed by signal re-enforcement, are sufficient to explain the second lineage decision [56]. Alternatively, it has been proposed that the origin of ICM cells influences their subsequent allocation to epiblast or PrE [28,69]. Live image tracing of embryos from early cleavage stages revealed a trend for the majority of cells becoming internalized during the fourth cell cycle to contribute to the epiblast, whereas those entering the ICM during the fifth or sixth cell cycle tended to generate PrE [28]. In another study, which used retrospective lineage tracing of fluorescent markers, no significant difference was observed between early and late entering cells [63]. The apparent controversy was resolved, as most discrepancies in the outcome were interpreted to originate from different experimental set-ups with both authors agreeing that ‘embryogenesis is a highly dynamic and regulative process with subtle trends that influence cell fate’ [70,71]. We support the notion that certain biases are most likely present in normal embryos, however any of these reported molecular lineage biases in mouse preimplantation development can readily be overridden by topological rearrangements for the first, and modulation of FGF signalling for the second lineage decision [49,62,63].

## 8. Naive pluripotency is acquired during epiblast specification and captured in embryonic stem cells

Naive pluripotency is the ability of a cell to self-renew while retaining the potential for unbiased differentiation and germline contribution in the context of normal development. Compelling evidence that ES cells are derived from the preimplantation epiblast was provided by Brook & Gardner [72], by means of micro-dissection of periimplantation embryos. Almost half of the epiblasts disaggregated and scattered over the culture well gave rise to one, two or occasionally three clonal ES cell lines. The fact that only a maximum of three clonal lines could be derived from a single preimplantation epiblast led to the speculation that maybe only a subpopulation of cells can give rise to an ES cell colony [72,73], suggesting that the property of naive pluripotency is not epiblast-wide. More recently, the use of two inhibitors (2i) in combination with leukaemia inhibitory factor (LIF) has allowed the derivation of ES cells from ‘recalcitrant’ mouse strains and rats [74–76]. PD0325901 mediates mitogen-activated protein kinase signalling inhibition, thereby eliminating auto-induced differentiation [77,78], while the glycogen synthase kinase 3 inhibitor CHIR99021 positively stimulates the biosynthetic capacity of ES cells and stabilizes β-catenin [79]. β-catenin has been shown to sequester a repressor of pluripotency genes, Tcf3 (Tcf7l1), from the nucleus, which stimulates expression of the naive pluripotency factors Esrrb, Nanog and Klf2 [80,81]. In 2i/LIF, ES cell derivation from the E4.5 blastocyst is very efficient. Dissociated ICMs at this stage have been shown to produce ES cell colonies from all embryos analysed with numbers of clones ranging from two to 12 [62], throwing into question the hypothesis that naive pluripotency is restricted to privileged cells within the epiblast [73].

Although ES cells are commonly derived from the blastocyst stage, they can be established from various preimplantation stages and even from single blastomeres [82–85]. The resultant ES cells have very similar characteristics, suggesting that, during derivation, they progress to a common developmental stage from which *in vitro* self-renewal can ensue. Single cell ES cell derivation from dissociated embryos from 8-cell to the early postimplantation egg cylinder stage in 2i/LIF on gelatin demonstrates that clonal ES cell lines can be derived efficiently only from mid- and late blastocyst stages [86]. This study further showed that during derivation, epiblast cells do not traverse through distinct developmental states at a transcriptional level and cluster with the preimplantation epiblast at all times [86]. Thus, the window of opportunity to capture the epiblast state *in vitro* coincides with the initiation of ICM heterogeneity and epiblast specification. This is further supported by the observation that clonal ES cell colony numbers strictly correlate with preimplantation epiblast cell numbers, which can be modulated by activation and inhibition of FGF signalling [62,86]. Collectively, this demonstrates that naive pluripotency is a state acquired during preimplantation development, rather than representing a refined derivative of totipotency.

Epiblast cells can self-renew *in vitro* and the foundation for this property may be rooted in their self-renewal ability *in vivo*. Diapause is a facultative condition of embryonic arrest in rodents and other species [87–89], which occurs when implantation is prevented by oestrogen deprivation caused by persistent suckling of a previous litter. This phenomenon can be mimicked experimentally by ovariectomy or administration of an oestrogen antagonist. In diapause, the embryo develops until the late blastocyst stage and segregates epiblast and PrE. Interestingly, diapause embryos were originally used to derive mouse ES cells [90] and have been shown to facilitate ES cell derivation in conventional culture conditions on feeders and in the presence of serum [72]. Quantification of inner cells from diapause embryos revealed a small but significant increase in ICM cell number, implying that the cells continue to proliferate [91]. The fact that diapause embryos retain their developmental potential suggests that mouse epiblast cells can undergo self-renewal *in vivo*.

**Figure 5.**
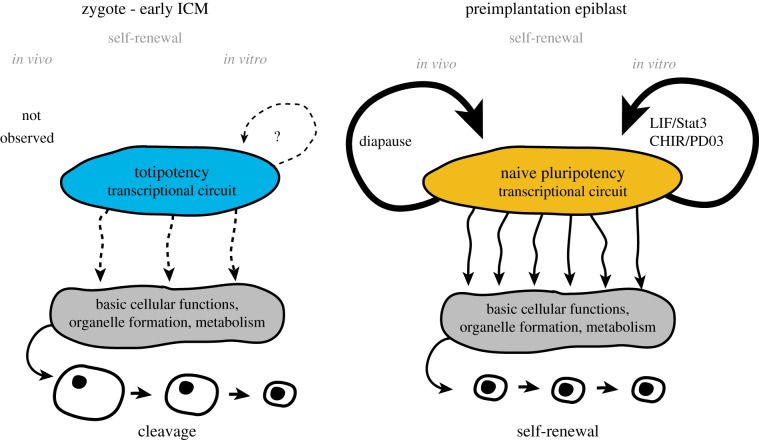
Representation of hypothetical totipotent and naive pluripotent transcriptional circuitries. Early embryonic cells from zygote to the early ICM stage strictly undergo cleavage and are unable to support self-sufficient proliferation. This may be caused by incomplete transcriptional interactions of totipotent circuitries with the basic housekeeping machinery. By contrast, cleavage ends at around the time of epiblast specification, thus rendering the preimplantation epiblast capable of cell-autonomous self-renewal. (Online version in colour.)

The transcriptional network of the initially totipotent developmental stages changes drastically after almost every cell division [20,92] and the common features of totipotency *in vivo* therefore remain ill defined. Developmentally, the closest totipotent state to naive pluripotency would be the early ICM, when the embryo is still undergoing cleavage [5]. We propose that the transcriptional networks operating during totipotent stages *in vivo* are incompletely connected to the basic cellular housekeeping machinery, including cell cycle checkpoints, and are thus incompatible with self-sufficiency and autonomous proliferation. By contrast, naive pluripotent epiblast cells have developed the capacity for cell-autonomous self-renewal *in vitro* and, during diapause, *in vivo* (figure 5). Currently, there are no culture conditions established to capture pure populations of authentic, self-renewing blastomeres or early ICM cells. Such totipotent cell lines would have to co-express both early epiblast and extraembryonic markers, readily differentiate into extraembryonic lineages *in vitro* within 48 h and efficiently contribute to both embryonic and extraembryonic tissues in chimaera assays. The establishment of self-renewing totipotent cells *in vitro* will strongly depend upon artificial integration of the totipotent transcriptional circuit to the housekeeping machinery. Moreover, it is likely that the temporal presence of maternal genes substantially contributes to a totipotent transcriptional network. Such key factors would have to be identified and expressed in a dosage and time-controlled manner in genetically engineered cells. In contrast to ES cells, self-renewing totipotent cells would lack a genuine embryonic counterpart and therefore it might be challenging, although theoretically possible, to generate such lines in the future.

## 9. Prerequisites for acquisition of epiblast identity

Early ICM cells co-express epiblast markers, such as Klf2, Sox2 and Nanog, and early PrE markers, including Gata6, Pdgfra and FGFR2 [20,56,86]. This delicate balance of opposing lineage specifiers sets the scene for complete lineage segregation within 24 h. Notably, this timing differs substantially from PrE-like differentiation from ES cells in both embryoid body [93] and monolayer [94] based protocols, in which robust PrE marker induction typically takes around 5 days or longer [94–96]. In presumptive epiblast cells, Nanog and Sox2 become upregulated and repress the sequential activation of the PrE specifiers [64,97,98].

Transcriptional differences during development would predict certain associated epigenetic motifs. Genome wide erasure of DNA-methylation is associated with naive pluripotency [99,100]. This resetting of epigenetic signatures is potentially crucial for unrestricted germ-layer differentiation. In females, the paternally inherited X-chromosome is silenced during the first round of cleavage divisions. Reactivation occurs transiently and exclusively in the embryonic lineage just before implantation [101]. Moreover, there is a correlation of the epigenetic status in epiblast cells in the embryo and ES cells *in vitro*. Electron spectroscopic imaging of early mouse development has shown that in morula and epiblast the chromatin is distributed as an extended meshwork of uncompacted fibres, indistinguishable from that of ES cells. By contrast, the chromosomes of extraembryonic lineages were found to be denser and more compacted [102]. This supports the notion that naive pluripotency is associated with an open chromatin state.

Another potential factor involved in epiblast specification may be the duration of occupation of an internal position and/or the exposure to extracellular matrix within the ICM. The early ICM expresses a very specific pattern of Laminin511 (Lama5, Lamb1, Lamc1), integrins and fibronectin [65,86]. Isolated early ICM cells can develop the properties of functional epiblast *in vitro*, when cultured on an attachment matrix consisting of Laminin511 and fibronectin in the presence of 2i/LIF [86]. The history of cell divisions in the preimplantation embryo may similarly contribute to the maturation of a self-sufficient, pluripotent founder cell population. Acquisition of epiblast or PrE fate is a gradual process [20,56,103]. The ability of isolated ICM cells to give rise to ES cell colonies *in vitro* appears to coincide with the departure of potential for inter-lineage conversion [86]. An intriguing possibility is that each ICM cell becomes irreversibly committed to either PrE or epiblast within a single cell cycle, most likely the seventh (figure 1). This may also coincide with the end of cleavage and the initiation of embryonic growth.

## 10. Exit from naive pluripotency *in vivo*

A major rearrangement of the epiblast occurs following implantation. From a loosely adherent ball of cells, a single-layered cup-shaped epithelium emerges. This process was long believed to occur as a result of apoptosis in the cells not in contact with the visceral endoderm in a BMP-dependent manner [104]. Recently, this hypothesis has been elegantly refuted and alternatively attributed to self-organizational behaviour of the epiblast [105]. During implantation, epiblast cells rearrange to form a rosette, probably due to basal membrane-stimulated integrin signalling. This establishment of apical–basal polarity is a prerequisite for lumenogenesis and subsequent gastrulation [105]. The transcriptional signature specific to the primed state of pluripotency includes downregulation of naive pluripotency markers such as Rex1, Klf2, Klf4, Tbx3 and Tfcp2l1 as well as upregulation of Pou3f1, Otx2 and FGF5 [86,106–108].

One of the key drivers of exit from naive pluripotency is FGF signalling. Preimplantation epiblast cells, and ES cells, their *in vitro* equivalent, autonomously drive progression of development by FGF4 expression [57,78]. Activation of the Erk-cascade directs transition to the early postimplantation epiblast, a tissue responsive to inductive cues for germ-layer specification and subsequent development. Furthermore, preimplantation epiblast cells express Nodal and upregulate Acvr2b upon implantation [86], which may facilitate the specification process. By contrast, the Wnt/Gsk3b signalling pathway has been implicated in maintenance of naive pluripotency [79–81,109]. Downregulation of Wnt/Gsk3b signalling is required for the transition from a naive to a primed state *in vitro* [79,109]. Interestingly, PrE cells express high levels of the Wnt inhibitor Dkk1 [86], potentially facilitating the pre- to postimplantation epiblast transition. However, recent work demonstrated that mice lacking the porcupine homologue Porcn (a protein required for acetylation and function of Wnt ligands) develop normally until gastrulation [110]. Further studies will be required to elucidate fully the complex role of Wnts, Gsk3b and β-catenin in preimplantation development.

Changes in signalling pathway activities between pre- and early postimplantation development are reflected in pluripotent stem cell lines derived from postimplantation epiblasts (EpiSCs), which exhibit distinct culture requirements from those of ES cells [106,107]. EpiSCs self-renew in the presence of FGF and Activin A, whereas ES cells differentiate upon activation of these pathways. Conversely, 2i-based culture conditions are detrimental for EpiSCs, suggesting that the ability to thrive in the absence of FGF signalling is a distinctive feature of mouse ES cells. In corroboration of this observation, the capacity for isolated epiblast cells to generate naive pluripotent cell lines in feeder-free 2i/LIF culture conditions is rapidly lost in the early postimplantation embryo [86], an event which functionally marks the exit from naive pluripotency *in vivo*.

## 11. Concluding remarks

The establishment of a pool of cells poised to respond to positional and signalling cues to form a highly complex organism is an elegant achievement of mammalian development. The first cell fates are specified by means of positional information, with an ‘inside’ requirement for the embryonic lineage. Cleavage continues and the inner cells set aside another extraembryonic lineage, subsequently required for patterning of the embryo. Towards the end of preimplantation development, the embryonic cells exit cleavage, a fundamental prerequisite for embryonic growth. At this time, the epiblast acquires the intriguing state of naive pluripotency, which can then be captured *in vitro* as self-renewing ES cells.
